# Associations Between Daily Step Counts and Sleep Parameters in Parkinson’s Disease: A Scoping Review

**DOI:** 10.3390/s25144447

**Published:** 2025-07-17

**Authors:** Tracy Milane, Edoardo Bianchini, Matthias Chardon, Fabio Augusto Barbieri, Clint Hansen, Nicolas Vuillerme

**Affiliations:** 1 AGEIS, Université Grenoble Alpes, 38000 Grenoble, France; tracymilane@gmail.com (T.M.); edoardo.bianchini@uniroma1.it (E.B.); matthias.chardon@univ-grenoble-alpes.fr (M.C.); 2 Department of Neurology, Kiel University, 24105 Kiel, Germany; 3 Department of Neuroscience, Mental Health and Sensory Organs (NESMOS), Sapienza University of Rome, 00189 Rome, Italy; 4 Department of Human Neurosciences, Sapienza University of Rome, 00185 Rome, Italy; 5 Human Movement Research Laboratory (MOVI-LAB), Department of Physical Education, School of Sciences, Sao Paulo State University (UNESP), Bauru 17033-360, SP, Brazil; fabio.barbieri@unesp.br; 6 Institut Universitaire de France, 75005 Paris, France

**Keywords:** Parkinson’s disease, sleep, sleep disorders, step count, wearable, activity monitor

## Abstract

**Background:** People with Parkinson’s disease (PwPD) often experience sleep disturbances and reduced physical activity. Altered sleep behavior and lower daily steps have been linked to disease severity and symptom burden. Although physical activity may influence sleep, few studies have examined the relationship between sleep parameters and daily steps in PD. This scoping review aimed to review current knowledge on sleep parameters and daily steps collected concurrently in PwPD and their potential association. **Methods:** A systematic search was conducted in five databases, PubMed, Web of Science, Sport Discus, Cochrane Library, and Scopus. Methodological quality was assessed using a customized quality checklist developed by Zanardi and collaborators for observational studies, based on Downs and Black’s work. **Results:** Out of 1421 records, five studies met the eligibility criteria and were included in the review. Four studies reported wearable-based measurements of both step count and sleep parameters, while one study reported wearable-based measurements of step count and self-reported sleep measures. Two studies examined the association between sleep parameters and step count. One study did not find any correlation between sleep and step count, whereas one study reported a positive correlation between daytime sleepiness and step count. **Conclusions:** This review highlighted the lack of research investigating the relationship between sleep parameters and step count as an indicator of physical activity in PwPD. Findings are inconsistent with a potential positive correlation emerging between daytime sleepiness and step count. Findings also pointed toward lower step count and reduced sleep duration in PwPD, as measured with wearable devices.

## 1. Introduction

Parkinson’s disease (PD) is a progressive neurodegenerative disorder that is a global health concern due to its increasing prevalence over the past decades [1]. PD is characterized by a wide range of motor and non-motor symptoms [2]. Motor impairments include rest tremor, rigidity, bradykinesia, and gait and balance impairment, as well as postural alterations [2]. These significantly hinder daily mobility in people with PD (PwPD) [3], often leading to lower physical activity levels [4].

In addition to motor symptoms, PwPD often experience non-motor symptoms (NMS), including depression and anxiety, olfactory deficits, sleep disturbances, urinary and gastrointestinal problems, autonomic failure, and cognitive impairments [5]. These symptoms can occur throughout the disease course and may have an even greater impact on quality of life than motor symptoms [6]. Sleep disturbances are often reported among the most troublesome NMS [6,7] and are highly prevalent among PwPD, significantly affecting both patients and their caregivers’ quality of life [8]. A recent study showed that PwPD with poor sleep quality experienced worse mobility, emotional well-being, activities of daily living, cognitions, communication, and bodily discomfort [9]. Sleep disturbances in PD include insomnia, rapid eye movement (REM) sleep behavior disorder (RBD), restless leg syndrome, sleep disordered breathing, and excessive daytime sleepiness [8]. Compared to age- and sex-matched healthy adults, PwPD exhibit altered sleep architecture, with reduced sleep efficiency and wake after sleep onset (WASO), as well as disrupted REM sleep [10].

Standard approaches for assessing sleep include sleep diaries and questionnaires (e.g., the Pittsburgh Sleep Quality Index (PSQI) or the Parkinson’s Disease Sleep Scale (PDSS)) [11]. However, these self-report measures of sleep are susceptible to recall and reporting bias [12]. To address these limitations, polysomnography and actigraphy are the two main objective methods to assess sleep in PD [13,14]. While polysomnography is considered the reference for sleep assessment [13], its implementation is challenging due to the need for trained personnel and the associated high costs. In contrast, wearable digital technology (e.g., wristbands, armbands, smartwatches, headbands, rings, clips, etc.) presents a potential solution for long-term monitoring in an unobtrusive way and in real-world conditions, providing easy access to extensive sleep data [15], for example, using flexible and stretchable sensors to detect body movements during sleep [16]. Actigraphy is a commonly used method to assess movement and sleep in non-laboratory settings [15]. A recent study by Matos et al. [17], encompassing 26 studies, highlights the growing interest in studying wearables for sleep in PwPD, and provides a comprehensive summary of the existing evidence on sleep monitoring under free-living conditions. However, wearable-based sleep assessment cannot assess sleep architecture and does not allow the collection of detailed sleep parameters compared to polysomnography. Moreover, although there are reports on the good correlation between polysomnography and actigraphy in healthy adults [18,19], this latter may over-/underestimate sleep metrics in adults with and without chronic conditions [20], in older women with insomnia [21], in patients with sleep disorders [22], and in PwPD [23].

Moreover, wearable devices offer the possibility of collecting additional parameters besides sleep data, such as mobility and physical activity metrics (e.g., step count or energy expenditure, sedentary behavior, etc.). Physical activity plays a crucial role in PD management, as it can help reduce symptoms severity [24] and some evidence also suggested a potential effect in slowing disease progression [25,26] while also alleviating NMS [27]. In terms of NMS, PwPD who engage in higher levels of daily physical activity experience better global cognition and lower levels of anxiety, apathy, and depression [28], as well as improved fatigue and sleep [29]. However, PwPD spend approximately 75% of their day in sedentary behavior [4], with their daily ambulatory activity often falling below the recommended levels. Furthermore, others NMS, such as cognitive impairment, excessive daytime sleepiness, depression, and fatigue, can also limit physical activity participation, leading to a more sedentary lifestyle [30].

Daily step count is an easy-to-collect and informative metric to study physical activity and real-world mobility [31] due to its simplicity; it is easily comprehensible and interpretable by a general population. Additionally, a number of studies highlighted that an increase in daily steps is linked to a reduction in all-cause mortality [32,33] and specific conditions such as cardiovascular disorders and cancer [34] and dementia [35]. Regarding PwPD, previous evidence indicated a lower number of daily steps compared with healthy individuals [36] and PwPD taking less than 4200 steps have been reported to have a 23-fold decrease in meeting physical activity recommendations [37]. Moreover, the number of daily steps has been associated with disease severity [38]. The growth of commercial wearable devices, such as smartphones and smartwatches, has made step counting even more accessible, offering a non-invasive and continuous method for monitoring physical activity and real-world mobility [39]. Additionally, wearables could serve as motivational tools, encouraging individuals to take more steps and remain active [40].

Previous research has explored the relationship between wearable-based daily step count and sleep parameters in older adults [41], demonstrating a significant association between daily steps and sleep quality. Specifically, a higher daily step count was associated with greater sleep efficiency, fewer nighttime awakenings, reduced WASO, and naptime [41]. Similarly, Hirata et al. [42] examined the association between sleep and physical activity in patients with chronic obstructive pulmonary disease using wearable devices, and reported that those who spent more than nine hours lying in bed experienced more fragmented sleep and a lower daily step count. Additionally, Vinod et al. [43] investigated the relationship between sleep quality and physical activity parameters in individuals with multiple sclerosis using wrist-worn sensors, revealing that the sleep regularity index and intra-day variability were associated with the duration of light and moderate physical activity. Similarly, Ophey et al. [44] examined the relationship between accelerometer-derived sleep and physical activity measures in PwPD, reporting that greater sleep regularity was associated with higher physical activity levels, and that physical activity correlated with more stable circadian rhythms. However, few studies have specifically investigated the relationship between sleep parameters and daily step count in PwPD.

Therefore, in this scoping review, we reviewed the current knowledge on sleep parameters, encompassing both subjective (e.g., questionnaires) and objective (i.e., wearable-based measures) measures, and daily steps collected concurrently in PwPD and their potential relationship.

## 2. Methods

### 2.1. Protocol and Registration

A scoping review was conducted to evaluate the existing knowledge and identify gaps for future research and interventions. The review’s protocol has been registered with the International Prospective Register of Systematic Reviews (PROSPERO) (registration number: CRD42024543782). The scoping review was conducted in accordance with the Preferred Reporting Items for Systematic Reviews and Meta-Analyses Extension for Scoping Reviews (PRISMA-ScR) guidelines [45].

### 2.2. Eligibility Criteria

The inclusion and exclusion criteria for the studies were determined using a population, intervention, comparison, outcome, and study design (PICOS) tool. This review included original articles published in English, French, German, Italian, or Portuguese in peer-reviewed scientific journals. Case reports, abstracts, editorials, letters to the editor, case studies, books, chapters, reviews, meta-analyses, and other grey literature materials (government reports, policy statements and issues papers, conference proceedings, preprints articles, theses, and dissertations) were excluded (study design). Eligible participants were adults aged 18 years or older, and diagnosed with PD. Studies involving children, adolescents, patients diagnosed with other types of parkinsonism, or animal models were excluded (population). We focused on studies that reported measurements of both daily step count and sleep parameters in PwPD (intervention). The inclusion and exclusion criteria using the PICOS tool are described in Table 1.

### 2.3. Data Sources and Search Strategy

PubMed, Web of Science, SPORTDiscus, Cochrane Library, and Scopus databases were systematically searched up to 19 December 2024. The search strategy focused on articles containing information related to three main topics: (1) Parkinson’s disease, (2) sleep disorders, and (3) steps, including all relevant subsets of these terms. Keywords for each topic were combined using the Boolean operators “AND” and “OR”. The final search strategy combined the three categories and was structured as follows: (“Parkinson Disease” OR “Parkinson’s disease” OR “Parkinson” OR “PD”) AND (“sleep” OR “insomnia” OR “dyssomnia”) AND (“step*” OR “physical activity” OR “physical inactivity” OR “sedentary” OR “sedentariness” OR “sitting” OR “seated” OR “Metabolic Equivalent” OR “exercise volume”). The search was restricted to titles, abstracts, and keywords.

### 2.4. Study Selection

After removing duplicate records, two independent reviewers (TM and MC) conducted the initial screening of the titles, abstracts, and keywords from each article retrieved in the electronic database searches to identify potentially relevant studies. Duplicate records were removed after completing the database search. Following this first screening, the same two reviewers assessed the full-text articles for eligibility based on the abovementioned criteria. Any disagreements during the screening process for inclusion/exclusion were resolved by a consensus through discussion or, if necessary, by consulting a third reviewer (NV).

### 2.5. Data Extraction

After completing the screening process, the same two reviewers (TM and MC) independently extracted data from each included study and cross-checked it for consistency. Any discrepancies between the two reviewers were resolved through consensus discussions, and if disagreement persisted, a third reviewer (NV) was consulted for the final decision. Data extraction followed a prebuilt table that included details on study characteristics, population demographics, and the main measures of sleep and daily step count in PwPD. Study characteristics referred to the authors’ names, title, year of publication, journal’s name, country of study, study design, funding source, and conflicts of interest. Population-related information included sample size, age, gender, weight, height, body mass index (BMI), disease duration and severity, occupational status, and education. The reported outcome measures for daily step counts and sleep included details about the measurement methods (questionnaires, wearable device used, its placement, wear duration, setting, and participant instructions) and key findings of daily step counts and sleep parameters. Additionally, conclusions and clinical or research implications were extracted. In cases of missing data, the study authors were contacted to provide additional information.

### 2.6. Methodological Quality

The methodological quality of the included studies was independently assessed by two reviewers (TM and MC). In cases of disagreement, a third reviewer (NW) was contacted to reach a consensus. The quality assessment was performed in accordance with a previous work from our group [46], using a customized quality checklist recently developed by Zanardi et al. [47], based on preliminary work by Downs and Black [48]. Originally designed for randomized and non-randomized intervention studies, the checklist was modified by removing some items to be relevant for observational studies.

## 3. Results

### 3.1. Selection of Studies

**Figure 1:**
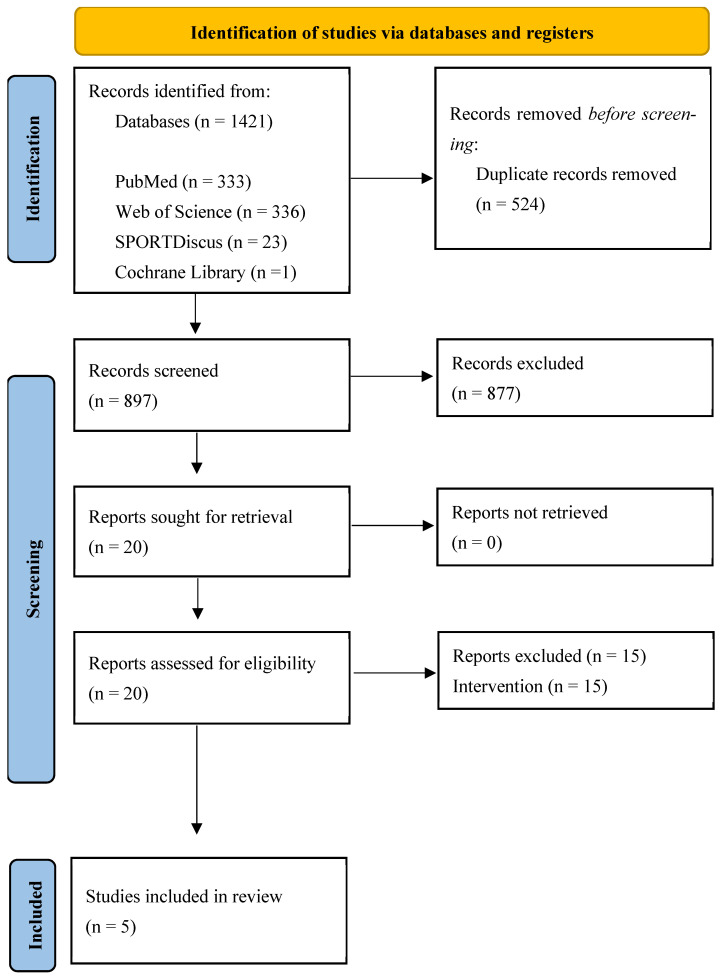
Flow diagram of the articles included in the review. The number of original articles is indicated at each stage of the search.

A total of 1421 records were initially identified through database searching: 333 from PubMed, 336 from Web of Science, 23 from Sport Discus, 1 from Cochrane, and 728 from Scopus. After removing duplicates (n = 524), a total of 897 unique records remained. After screening titles, abstracts, and keywords, 20 full texts were read and assessed for eligibility. After full text-reading, 15 studies were excluded due to the intervention and five studies met our eligibility criteria and were included in this review [49,50,51,52,53]. Among them, four studies [49,51,52,53] reported objective measurements of both daily step count and sleep parameters using wearable technologies, while one study [50] reported objective measurements of daily step count using wearable technologies and subjective assessments of sleep parameters. Figure 1 presents the process of the study selection.

### 3.2. Methodological Quality

All studies [49,50,51,52,53] clearly described the hypothesis/aim/objective, reported the main findings, showed random variability in the data, described probability values, measured the appropriate statistic, and had valid outcome measures. Four out of five studies (80%) [50,51,52,53] clearly described the main outcomes and the characteristics of the participants. Two studies (40%) [52,53] listed the principal confounders, while two studies (40%) [50,51] listed them partially. Three studies (60%) [49,50,52] recruited the participants of the groups in the same period. Quality assessment details are presented in Table 2.

### 3.3. Study Characteristics

The publication year of the included studies ranged from 2020 to 2024. The total number of PwPD across all studies was 429, with sample sizes ranging from 25 [51] to 149 [49] participants. Study characteristics are presented in Table 3.

### 3.4. Sample Characteristics

Table 4 provides demographic, anthropometric, and clinical characteristics of the participants.

### 3.5. Data Collection

Table 5 provides characteristics of the various wearable devices used in the included studies.

All studies [49,50,51,52,53] assessed daily step count using wearable devices. Sleep parameters were assessed with wearable devices in four studies (80%) [49,51,52,53], one of which also used questionnaires [49]. Additionally, one study (20%) [50] relied solely on questionnaires for sleep assessment.

#### 3.5.1. Wearable Devices Used

Schalkamp et al. [49] utilized data from the Parkinson’s Progression Monitoring Initiative (PPMI) cohort, collected using the Verily Study Watch (Verily Life Sciences LLC, South San Francisco, CA, USA). This multi-sensor, wrist-worn smartwatch is equipped with an accelerometer, a gyroscope, electroencephalography (EEG), and photoplethysmography (PPG), and enables passive data collection during daily activities. Two studies (40%) [52,53] used the SenseWear Arm Band (SWA) activity monitor (BodyMedia, Inc., Pittsburg, PA, USA), a biaxial device worn on the triceps of the dominant upper limb with an elastic band. In the study by Prusynski et al. [51], participants wore a commercially available activity monitor (Fitbit Charge HR, Fitbit Inc., San Francisco, CA, USA) on their non-dominant wrist. In the study from Adams et al. [50], participants wore an Apple Watch (Series 4 or 5) on their more-affected side. In the included studies, wearable devices were used solely for monitoring step counts or sleep parameters and were not employed as part of any intervention aimed at improving these outcomes.

#### 3.5.2. Data Collection Period

In Schalkamp et al. [49], data were collected between 2018 and 2020. Step count data spanned an average of 1.25 ± 0.54 years, with a mean recorded duration of 0.91 ± 0.52 years. Sleep data covered an average of 1.19 ± 0.57 years, with a mean recorded duration of 8.4 ± 6.68 days. Data retrieval occurred in November 2022, resulting in an average of 485 days of home monitoring [49]. In the two studies by Aktar and collaborators [52,53], participants were instructed to wear the SWA for seven consecutive days at home, removing it only during water-related activities. However, the exact final wearing time period was not reported [52,53]. In Prusynski et al. [51], participants were asked to wear the monitor continuously for 14 days and 14 nights, removing it only for charging or during water-related activities. On average, the device was not worn for 5% of the total 14-day period in the PwPD group, and 6% in the healthy older adults group [51]. In Adams et al. [50], data were collected for at least one week for six times, following in-person visits.

#### 3.5.3. Wearable-Based Step and Sleep Outcomes

The Verily Study Watch collected the hourly step count and sleep parameters (sleep efficiency, the number of awakenings, total sleep time, WASO, total NREM time, total REM time, and total deep NREM time). The SWA measured daily step counts (steps per week) and sleep duration (minutes per week) [52,53]. In Aktar, Balci et al. [52], step count data were used to categorize PwPD participants as sedentary and non-sedentary, with those taking fewer than 5000 steps per day classified as sedentary. The Fitbit Charge HR recorded daily step count, nighttime sleep variables (total nighttime sleep in minutes, the number of awakenings, and WASO in minutes), and daytime sleep variables (total daytime sleep in minutes and the number of naps) [51]. The Apple Watch (Series 4 or 5) recorded the number of steps per hour and per day [50].

#### 3.5.4. Questionnaire-Based Sleep Outcomes

In addition to wearable-based data collection, sleep was assessed using two questionnaires: the REM sleep behavior disorder screening questionnaire (RBDSQ), to evaluate the presence of RBD, and the Epworth Sleepiness Scale (ESS), to evaluate daytime sleepiness [49,50].

### 3.6. Sleep and Daily Step Count Main Outcomes

#### 3.6.1. Wearable-Based Step Count Measurements and Self-Reported Sleep Assessments

Sleep and step count results are summarized in Table 6. PwPD had significantly higher RBDSQ scores than controls, with a 63% higher score at baseline and an 80% increase at the month 12 visit (month 12 visit: PD: 4.5 ± 3.2 vs. control: 2.5 ± 2.1, *p* < 0.001) [50]. However, no significant differences were found in ESS scores between PwPD and controls at either time point [50].

A significant positive correlation was observed between ESS scores and hourly step count (r = 0.314; *p* value = 0.006) [49], indicating that higher levels of daytime sleepiness were associated with increased steps per hour (Table 7). This result remains significant after applying the FDR correction (*p* corrected FDR = 0.046), further validating that PwPD who experience greater daytime sleepiness tend to take more steps [49]. In contrast, no significant correlation was found between RBDSQ scores and hourly step count [49].

#### 3.6.2. Wearable Device-Based Measurements of Step Count and Sleep Parameters

PwPD experienced reduced sleep duration compared to healthy controls in two studies [51,53]. Prusynski et al. [51] reported that PwPD (n = 25) slept on average 75 min less per night, representing an 18% reduction compared to healthy older adults (n = 27) (347 ± 108 min vs. 422 ± 41 min, *p* < 0.01). Furthermore, although daytime sleep duration was similar between groups, PwPD tended to take more frequent naps. The number of awakenings and WASO were comparable between groups [51]. Additionally, Aktar, Donmez Colakoglu et al. [53] reported significantly shorter weekly sleep duration in PwPD (n = 56) compared to healthy subjects (n = 58) (2598.50 [IQR: 1950.75–2947.00] minutes vs. 2760.50 [IQR: 2515.75–3196.75] minutes, *p* < 0.05). In a second study by Aktar, Balci et al. [52], while the sedentary PD group (n = 25) had longer sleep duration compared to the non-sedentary PD group (n = 35), this difference was not statistically significant (6.55 ± 1.90 vs. 5.99 ± 1.71, *p* > 0.05, increase by 9%).

PwPD also exhibited lower step counts compared to healthy controls in three studies (60%) [50,51,53]. Prusynski et al. [51], reported that PwPD (n = 25) walked significantly less, taking an average of 5792 fewer steps per day, representing a 49% reduction compared to healthy older adults (n = 27) (5953 ± 2365 vs. 11,745 ± 3891, *p* < 0.001). Similarly, Aktar, Donmez Colakoglu et al. [53] found that PwPD (n = 56) had lower step counts compared to healthy subjects (n = 58) (51,854.50 [IQR: 36,724.50–62,772.00] vs. 35,606.50 [IQR: 24,766.50–51,020.25], *p* < 0.05). Adams et al. [50] found that compared to control (n = 50), PwPD (n = 82) walked significantly fewer steps per hour at baseline (238 ± 129 vs. 362 ± 214, *p* < 0.001).

Wearable-based sleep parameters (e.g., sleep efficiency, awakenings, total sleep time, WASO, NREM, REM, and deep NREM) showed no significant association with hourly step counts in people with Parkinson’s disease (PwPD), as measured by the Verily Study Watch [49]. This finding is consistent with Prusynski et al. [51], who also reported no significant relationship between average nighttime sleep duration and daily step counts in both PwPD and healthy older adults (see Table 7).

## 4. Discussion

Overall, no consistent correlation between daily step counts and sleep parameters in PwPD were found, although the evidence is limited. Notably, one study reported a positive correlation between daytime sleepiness (ESS scores) and hourly step counts in PwPD, meaning those with greater sleepiness paradoxically showed more physical activity [48]. This is counterintuitive, as excessive daytime sleepiness typically associates with reduced activity [54,55].

Conversely, compared to healthy older adults, PwPD consistently exhibited significantly worse sleep parameters [51,53] and reduced step counts [50,51,53].

Medications likely play a role in the unexpected link between increased sleepiness and higher activity. Antiparkinsonian medications can cause excessive daytime sleepiness due to sedative effects [56]. Simultaneously, drugs like levodopa improve motor symptoms, potentially increasing mobility despite increased sleepiness [57]. This dual effect could explain why individuals experiencing more sleepiness might also exhibit greater movement. A similar compensatory movement pattern in response to poor sleep has been observed in people with multiple sclerosis, where greater sleep variability was linked to increased physical activity [43], suggesting this might be a broader response in chronic neurological conditions.

Those findings contrast with a study in healthy office employees (aged 25–35 years) where increased daily steps significantly reduced daytime sleepiness [58]. This divergence is likely due to the profound differences in age, health status, and clinical characteristics between PwPD and healthy adults. In PwPD, increased steps could potentially increase fatigue, leading to greater daytime sleepiness. Alternatively, both excessive daytime sleepiness and increased steps might stem from nighttime sleep disturbances, prompting more activity during the day.

While one study in PwPD found no correlation between REM sleep behavior disorder (RBDSQ scores) or other sleep parameters (e.g., sleep duration and efficiency) and hourly step count [49], previous research in community-dwelling older adults showed that higher daily steps correlated positively with sleep efficiency and negatively with wakefulness after sleep onset (WASO), awakenings, and naptime [41]. An inverted U-shaped curve has been proposed, suggesting both very low and very high activity levels can negatively impact sleep quality and duration [58]. Regular physical activity, through mechanisms like endorphin release, serotonin and norepinephrine stimulation, and the activation of serotonergic and GABAergic neurons, can improve sleep quality, reduce sleep latency, and alleviate stress and anxiety [59,60]. Physical activity may also increase brain-derived neurotrophic factor (BDNF) levels, enhancing slow-wave sleep [60].

The reduced sleep duration observed in PwPD via wearables [51,53] aligns with existing literature [13,14,61]. PwPD often exhibit shorter nighttime sleep and high sleep fragmentation, potentially due to muscle cramps, restless leg syndrome, and increased muscle tension [62,63,64]. Short sleep duration (<5 h) is also linked to worse quality of life in PwPD, especially in advanced stages with sleep disorders [65]. While total daytime sleep did not differ significantly, PwPD tended to nap more frequently, suggesting a compensatory mechanism for poor nighttime sleep [51]. Sleep disturbances in PD are multifactorial, involving neurodegeneration, neurotransmitter imbalances, and the impact of antiparkinsonian therapies [14,62]. Furthermore, PwPD frequently experience REM sleep behavior disorder (RBD), which is associated with poor sleep quality due to disruptive motor symptoms and distressing dreams [14,50].

PwPD also exhibit reduced step counts [50,51,53], likely due to gait impairments and bradykinesia [66,67]. Interestingly, one study found no significant difference in sleep duration between sedentary and non-sedentary PwPD, though sedentary individuals tended to have longer sleep [52]. While increased step count is a protective factor for cognitive function and decreases stress and daytime sleepiness [41,58], one study in PwPD found no significant association between nighttime sleep and daily steps, but a negative association with sedentary time: greater sleep duration correlated with reduced sedentary time [51]. This highlights the importance of considering other aspects of physical activity, such as duration and intensity, beyond just step count.

## 5. Limitations

This scoping review highlights significant gaps in the current literature on the relationship between sleep and physical activity in PwPD, as measured by wearable devices. Only five studies were included in this review. A major reason for excluding others was their failure to concurrently evaluate both step count and sleep using wearable devices. Since most wearables can monitor both, future studies should prioritize collecting both sleep and step data simultaneously rather than focusing on only one. While continuous data collection impacts battery life, recent evidence suggests four consecutive days are sufficient for reliable step counts with commercial smartwatches [68]. However, there is a need to determine the minimum number of days required for reliable wearable-based sleep data to confirm the feasibility of a one-week monitoring period for both parameters.

Some past studies may have collected both sleep and step data but did not analyze their relationship. Reanalyzing raw data from these prior studies could significantly expand our understanding of sleep–activity interplay in PwPD, promoting a more frugal research model by maximizing existing data and reducing redundant collection efforts.

All included studies treated sleep and step count as independent variables, with only two examining their relationship [49,51]. Furthermore, studies had relatively small sample sizes, ranging from 25 to 149 participants. The diverse wearable devices (e.g., the Verily Study Watch, Fitbit Charge HR, and Apple Watch) and methodologies used to assess sleep and step count limit comparability across findings. Device placement also varied (wrist vs. triceps), which can influence measurements [69,70,71] and the accelerometer type (e.g., biaxial vs. triaxial) can affect the accuracy of step counts and the reliability of various sleep parameters.

While Fitbits can accurately measure total sleep time, they are less precise for other sleep parameters like sleep efficiency [51]. Wearables also have limitations in assessing sleep architecture and stages, which are crucial for diagnosing and treating sleep disorders [72]. For instance, some devices cannot differentiate between deep and light sleep [73]. Additionally, step count alone does not capture key aspects of functional mobility, such as activity intensity, type, or specific gait parameters (e.g., stride length and walking speed) [49].

Two studies were cross-sectional [52,53], preventing the determination of causal relationships. A significant limitation across studies was the inadequate reporting of key confounders, such as BMI, disease duration, disease stage, and medication use. This omission hinders the ability to control for factors that can influence both sleep and physical activity. For example, dopaminergic medications can improve motor function while simultaneously affecting sleep, confounding the relationship. Similarly, PwPD in advanced stages might experience both poorer sleep and reduced mobility. A lack of reporting on participant recruitment (e.g., whether participants came from the same population or recruitment period) also introduces risks of selection and temporal biases, potentially reducing the validity of findings.

These limitations underscore the critical need for better reporting of recruitment methods and participant characteristics in future studies to improve the validity and comparability of research on sleep and physical activity in PwPD.

## 6. Conclusions

This scoping review underscores a significant gap in research exploring the link between sleep parameters and step counts in PwPD. Existing studies present inconsistent findings, though a curious positive correlation between daytime sleepiness and step count occasionally emerges. Notably, only two studies have actually investigated this correlation, with just one reporting the unexpected positive association between daytime sleepiness and step counts. The limited available evidence suggests PwPD tend to have lower step counts and reduced sleep duration when measured by wearable devices.

To address these shortcomings, future research must delve deeper into the relationship between step count and sleep parameters using wearable technology. Importantly, longitudinal designs are crucial to capture how these variables change over time, moving beyond the limitations of cross-sectional studies. While the heterogeneity of protocols across the five included studies prevents us from recommending standardized wearable procedures, recent findings suggest that as few as four days of data collection might be enough for reliable daily step counts using commercial smartwatches [68]. When choosing devices, tri-axial accelerometers are preferable due to their superior accuracy in detecting subtle changes in walking behavior and identifying steps [74,75].

To gain a more complete understanding of the interplay between sleep and physical activity in PwPD, future studies should extend beyond mere total step counts. They should incorporate measures like activity intensity, sedentary behavior, and the frequency and duration of activity bouts. Similarly, future research should prioritize objective sleep measures such as sleep efficiency, which offers insights into both sleep quantity and fragmentation, and total sleep time, given its established links to poor quality of life [65] and increased risk of all-cause mortality [76].

## Figures and Tables

**Table 1:** Eligibility criteria of the included studies using PICOS.

	Inclusion Criteria	Exclusion Criteria
**Population**	People with Parkinson’s disease	Children, adolescents, patients diagnosed with other types of parkinsonism, or animal models
**Intervention**	Measurements of daily step counts and sleep parameters	None
**Comparator**	Not applicable	Not applicable
**Outcomes**	Any measure quantifying daily step counts and sleep parameters (e.g., correlation coefficients)	None
**Study design**	Original articles published in English, French, German, Italian, or Portuguese in a peer-reviewed journal.	Case reports, abstracts, editorials, letters to the editor, case studies, books, chapters, reviews, meta-analyses, and other grey literature materials (government reports, policy statements and issues papers, conference proceedings, preprints articles, theses, and dissertations).

**Table 2:** Quality assessment of included studies based on selected items of a customized quality checklist recently developed by Zanardi and colleagues [47].

Study	Quality Index Item Number													Total
	1	2	3	5	6	7	10	11	12	18	20	21	22	
Aktar et al., 2020 [53]	1	1	1	2	1	1	1	0	0	1	1	0	0	10
Aktar et al., 2020 [52]	1	1	1	2	1	1	1	0	0	1	1	1	1	12
Prusynski et al., 2022 [51]	1	1	1	1	1	1	1	0	0	1	1	0	0	9
Adams et al., 2024 [50]	1	1	1	1	1	1	1	0	0	1	1	0	1	10
Schalkamp et al., 2024 [49]	1	0	0	0	1	1	1	0	0	1	1	0	1	7
%	100	80%	80%	60%	100%	100%	100%	0	0	100%	100%	20%	60%	

**Table 3:** Characteristics of the included studies.

First Author, Year	Country	Title	Journal	Objective	Funding Source	Design
Aktar et al., 2020 [53]	Turkey	Does the postural stability of patients with Parkinson’s disease affect the physical activity?	*International Journal of Rehabilitation Research*	To examine the physical activity levels in patients with Parkinson’s disease, compared with healthy subjects, and their association with postural stability.	Not reported.	Cross-sectional study
Aktar et al., 2020 [52]	Turkey	Physical activity in patients with Parkinson’s disease: A holistic approach based on the ICF model	*Clinical Neurology and Neurosurgery*	(1) To compare the effect of biopsychosocial factors based on ICF (international classification of functioning, disability, and health) domains in sedentary and non-sedentary PD patients. (2) To investigate the association between physical activity level and biopsychosocial factors within sedentary and non-sedentary PD patients.	This research did not receive any specific grant from funding agencies in the public, commercial, or not-for-profit sectors.	Retrospective subgroup analysis of a previously published cross-sectional design
Prusynski et al., 2022 [51]	USA	The association between sleep deficits and sedentary behavior in people with mild Parkinson disease	*Disability and Rehabilitation*	To use a commercially available activity monitor to examine the association between sleep and physical activity in participants with mild PD and in healthy older adults.	The Institute of Translational Health Sciences at the University of Washington under Grant UL1TR002319. The National Institutes of Health under Grant NICHD/NCMRR K01HD076183.	Secondary analysis of a prospective observational study
Adams et al., 2024 [50]	USA	Using a smartwatch and smartphone to assess early Parkinson’s disease in the WATCH-PD study over 12 months	*npj Parkinson’s Disease*	“We evaluated the longitudinal change in these assessments over 12 months in a multicenter observational study using a generalized additive model, which permitted flexible modeling of at-home data”.	Biogen, Takeda, and the members of the Critical Path for Parkinson’s Consortium 3DT Initiative, Stage 2. Innovation in Regulatory Science Award from the Burroughs Wellcome Fund.	Multicenter longitudinal observational study
Schalkamp et al., 2024 [49]	United Kingdom	Digital outcome measures from smartwatch data relate to non-motor features of Parkinson’s disease	*npj Parkinson’s Disease*	We used rich multi-modal data from the Parkinson’s disease Progression Marker Initiative (PPMI) cohort to investigate how standard digital outcome measures of physical activity, sleep, and vital signs obtained from passively collected free-living smartwatch data relate to clinically assessed non-motor signs and symptoms and evaluated their potential utility in the context of clinical care.	PPMI—a public-private partnership—is funded by the Michael J. Fox Foundation for Parkinson’s Research and funding partners, including 4D Pharma, Abbvie, AcureX, Allergan, Amathus Therapeutics, Aligning Science Across Parkinson’s, AskBio, Avid Radiopharmaceuticals, BIAL, Biogen, Biohaven, BioLegend, BlueRock Therapeutics, Bristol-Myers Squibb, Calico Labs, Celgene, Cerevel Therapeutics, Coave Therapeutics, DaCapo Brainscience, Denali, Edmond J. Safra Foundation, Eli Lilly, Gain Therapeutics, GE HealthCare, Genentech, GSK, Golub Capital, Handl Therapeutics, Insitro, Janssen Neuroscience, Lundbeck, Merck, Meso Scale Discovery, Mission Therapeutics, Neurocrine Biosciences, Pfizer, Piramal, Prevail Therapeutics, Roche, Sanofi, Servier, Sun Pharma Advanced Research Company, Takeda, Teva, UCB, Vanqua Bio, Verily, Voyager Therapeutics, the Weston Family Foundation, and Yumanity Therapeutics.	Longitudinal study

**Table 4:** Participants’ descriptive characteristics in included studies.

First Author, Year	Sample Size and Sex [M (n, %)]	Age (Years)	Anthropometric Measures (Height, m; Weight, kg; and BMI, kg/m^2^)	Disease Duration (Years)	H&Y Scale	MDS-UPDRS	Medication (LEDD, mg)	Medication State
Aktar et al., 2020 [53]	PD: n = 56, M: 34 (60.7%) Control: n = 58, M: 33 (56.9%)	PD: 66.00 (59.50–71.75) Control: 63.50 (57.75–69.25)	PD: Height: 1.66 (1.59–1.74) Weight: 78.50 (71.00–88.50) BMI: 28.54 (25.93–30.84) Control: Height: 1.67 (1.59–1.73) Weight: 78.00 (64.75–85.00) BMI: 27.59 (24.89–29.47)	5.00 (2.00–8.00)	2.00 (2.00–2.50)	MDS-UPDRS II: 8.50 (4.12–11.00) MDS-UPDRS III: 24.00 (17.00–29.75) Rigidity: 3.00 (2.00–5.00) Rest tremor: 1.00 (0.00–2.00)	478.50 (343.75–737.50)	On
Aktar et al., 2020 [52]	Sedentary PD: n = 25, M: 15 (60.0%) Non-sedentary PD: n = 35, M: 24 (68.6%)	Sedentary PD: 67.52 ± 7.24 Non-sedentary PD: 64.77 ± 6.85	Sedentary PD: Height: 1.67 ± 0.10 Weight: 79.28 ± 12.11 BMI: 28.34 ± 2.71 Non-sedentary PD: Height: 1.67 ± 0.09 Weight: 80.06 ± 11.66 BMI: 28.51 ± 3.70	Sedentary PD: 5.72 ± 4.25 Non-sedentary PD: 5.22 ± 4.02	Sedentary PD: 2.18 ± 0.65 Non-sedentary PD: 2.00 ± 0.64	Sedentary PD: MDS-UPDRS II: 8.90 ± 4.98 MDS-UPDRS III: 4.84 ± 9.33 Non-sedentary PD: MDS-UPDRS II: 7.20 ± 4.26 MDS-UPDRS III: 23.74 ± 10.09	Sedentary PD: 620.50 ± 364.45 Non-sedentary PD: 541.85 ± 301.01	On
Prusynski et al., 2022 [51]	PD: n = 25, M: NR HOA: n = 27, M: NR	PD: 69 ± 6 HOA: 67 ± 5	NR	NR	Median: 1	Total: 28 ± 16 MDS-UPDRS III: 12 ± 9	NR	NR
Adams et al., 2024 [50]	Baseline: PD: n = 82, M: 46 (56%) Control: n = 50, M: 18 (36%) Completed month 12 visit: PD: n = 57, M: 32 (56%) Control: n = 49, 18 (37%)	Baseline: PD: 63.3 ± 9.4 Control: 60.2 ± 9.9 Completed month 12 visit: PD: 64.1 ± 9.4 Control: 61.5 ± 9.7	NR	Baseline: 0.83 ± 0.61 Completed month 12 visit: 1.84 ± 0.61	n (%): Baseline: stage 0: 0 (0) stage 1: 19 (23) stage 2: 62 (76) stage 3–5: 1 (1) Completed month 12 visit: Stage 0: 0 (0) Stage 1: 7 (12) Stage 2: 49 (86) Stage 3–5: 1 (2)	Baseline: PD: Total: 35.2 ± 12.4 MDS-UPDRS I: 5.5 ± 3.6 MDS-UPDRS II: 5.6 ± 3.8 MDS-UPDRS III: 24.1 ± 10.2 Control: Total: 5.9 ± 5.3 MDS-UPDRS I: 2.8 ± 2.6 MDS-UPDRS II: 0.4 ± 1.0 MDS-UPDRS III: 2.7 ± 3.5 Completed month 12 visit: PD: Total: 40.5 ± 14.2 MDS-UPDRS I: 5.9 ± 4.0 MDS-UPDRS II: 7.1 ± 4.7 MDS-UPDRS III: 27.4 ± 11.1 Control: Total: 6.4 ± 5.0 MDS-UPDRS I: 3.0 ± 3.5 MDS-UPDRS II: 0.4 ± 1.1 MDS-UPDRS III: 2.9 ± 3.3	NR	Off at baseline
Schalkamp et al., 2024 [49]	n = 149, M: NR	67.69 ± 7.54	NR	NR	<3 at baseline	NR	NR	Off at baseline

Values are mean ± standard deviation, median (interquartile range), or number (%). PD: Parkinson’s disease; M: male; NR: not reported; H&Y: Hoehn and Yahr scale; MDS-UPDRS: Movement Disorder Society-Unified Parkinson’s Disease Rating Scale; LEDD: levodopa equivalent daily dose; and HOA: healthy older adults.

**Table 5:** Characteristics of the wearable devices.

Study	Sensor Name	Manufacturer	Sensor Type	N of Sensor	Wearing Location	Side	Duration	Setting	Wear Time
Aktar et al., 2020 [53]	SenseWear Arm Band activity monitor	BodyMedia, Inc., Pittsburg, PA, USA	Biaxial accelerometer	1	Triceps (upper extremity)	Dominant	7 consecutive days	Home	Continuously except during showering or swimming.
Aktar et al., 2020 [52]	SenseWear Arm Band activity monitor	BodyMedia, Inc., Pittsburg, PA, USA	Biaxial accelerometer	1	Triceps (upper extremity)	Dominant	7 consecutive days	Home	Continuously except during showering or swimming.
Prusynski et al., 2022 [51]	Fitbit Charge HR	FitbitInc., San Francisco, CA, USA	Triaxial accelerometer	1	Wrist	Non-dominant	14 days and 14 nights	Home	Continuously except for the time needed to charge the device and during water-related activities. The average percentage of time during the 14-day period that the device was not worn was 6% in the HOA group and 5% in the PD group.
Adams et al., 2024 [50]	Apple Watch 4 or 5	Apple, Inc., Cupertino, CA, USA	Triaxial accelerometer	1	Wrist	More affected side	12 months (for at least 1 week after each in-person visit (6 in-person visits))	Home	PD: an average of 14.4 h/day. Control: an average of 13.5 h/day.
Schalkamp et al., 2024 [49]	Verily Study Watch	Verily Life Sciences LLC, South San Francisco, CA, USA	Triaxial accelerometer	1	Wrist	Not reported	A mean of 485 days	Home	Not reported.

**Table 6:** Results of step count and sleep parameters in the included studies.

Author, Year	Outcome Measure	Statistical Analysis	Significance	Results (Mean ± SD or Median (IQR)) or Direction of Difference (↑↓) with Absolute Value
Aktar et al., 2020 [53]	Number of steps (steps/week) Sleep duration (minutes/week)	Mann–Whitney U Test	***p* < 0.05** ***p* < 0.05**	Number of steps: **↓ (16,248; 31%) PD group: 35,606.50 (24,766.50–51,020.25) vs. healthy control group: 51,854.50 (36,724.50–62,772.00)** Sleep duration: **↓ (162; 6%) PD group: 2598.50 (1950.75–2947.00) vs. healthy control group: 2760.50 (2515.75–3196.75)**
Aktar et al., 2020 [52]	Number of steps (steps/day) Sleep duration (hours/day)	Mann–Whitney U test	NS: *p* > 0.05	Sleep duration: **↑** Sedentary PD group: median (IQR): 6.58 (5.67–7.40), mean ± SD: 6.55 ± 1.90 vs. non-sedentary PD group: median (IQR): 5.69 (4.55–7.37), mean ± SD: 5.99 ± 1.71
Prusynski et al., 2022 [51]	Number of steps (steps/day) Nighttime sleep: Total nighttime sleep (minutes) Number of Awakenings Wake time after sleep onset (minutes) Daytime sleep: Total daytime sleep (minutes) Number of naps	Wilcoxon rank-sum tests	***p* < 0.001** ***p* < 0.01** NS: *p* = 0.50 NS: *p* = 0.56 NS: *p* = 0.12 NS: *p* = 0.07	Number of steps: **↓ (5792; 49%) PD group: 5953** ± **2365 vs. HOA group: 11 745** ± **3891** Total nighttime sleep: **↓ (75; 18%) PD group: 347** ± **108 vs. HOA group: 422** ± **41** Number of Awakenings: PD group: 1.9 ± 1.3 vs. HOA group: 2.2 ± 1.32 Wake time after sleep onset: PD group: 5.2 ± 3.4 vs. HOA group: 6.0 ± 4.0 Total daytime sleep: PD group: 112 ± 129 vs. HOA group: 71 ± 135 **↑** Number of naps: PD group: 1.3 ± 1.6 vs. HOA group: 0.6 ± 1.2
Adams et al., 2024 [50]	Number of steps (steps/day; steps/hour) RBDSQ ESS	Pairwise comparisons	NS: *p* = 0.13 ***p* < 0.001** NS: *p* = 0.16 NS: *p* = 0.29 ***p* < 0.001** ***p* < 0.001** NS: *p* = 0.66 NS: *p* = 0.50	Number of steps: Steps/day: Baseline: **↓** PD: 3494 ± 1930 vs. Control: 4930 ± 3270 Steps/hour: Baseline: **↓ (124; 34%) PD: 238 ± 129 vs. control: 362 ± 214** Steps/day: PD (n = 10): **↓** Baseline: 3052 ± 1306 vs. at month 12: 2331 ± 2010 Steps/hour: PD (n = 10): **↓** Baseline: 198 ± 82 vs. at month 12: 159 ± 142 RBDSQ: **Baseline:** **↑ (1.7; 63%) PD: 4.4 ± 3.1 vs. control: 2.7 ± 2.0** **Completed month 12 visit:** **↑ (2; 80%) PD: 4.5 ± 3.2 vs. control: 2.5 ± 2.1** ESS: Baseline: PD: 4.9 ± 3.2 vs. control: 4.6 ± 3.7 Completed month 12 visit: PD: 4.8 ± 2.5 vs. control: 4.4 ± 3.4

↑: increase in values in the first group compared to the second group; ↓: decrease in values in the first group compared to the second group; In bold: significant results; NS: not significant; PD: Parkinson’s disease; HOA: healthy older adults; RBDSQ: Rapid eye movement sleep Behavior Disorder Screening Questionnaire; ESS: Epworth Sleepiness Scale.

**Table 7:** Results of the Pearson correlation analysis and linear regression between step and sleep parameters.

		Sleep Parameters										
		Sleep Scales		Wearable Device								
		ESS	RBDSQ	Total Sleep Time (Hours)	Sleep Efficiency	Number of Awakenings	Wake After Sleep Onset Hour	Total NREM Time (Hours)	Total REM Time (Hours)	Total Deep NREM Time (Hours)	Total Light NREM Time (Hours)	30 min Additional Nighttime Sleep
**Step Count: Wearable Device**	**Step Count Total (Hours)**	**r = 0.313678 (*p* value = 0.006** **FDR corrected** ***p* = 0.046)**	r= 0.030652 (*p* value = 0.794 FDR corrected *p* = 0.891)	r = 0.339985 (*p* value = 0.042 FDR corrected *p* = 0.154)	r = 0.320028 (*p* value = 0.057 FDR corrected *p* = 0.190)	r = −0.12816 (*p* value = 0.456 FDR corrected *p* = 0.722)	r = −0.30702 (*p* value = 0.068 FDR corrected *p* = 0.220)	r = 0.345606 (*p* value = 0.0389 FDR corrected *p* = 0.147)	r = 0.193733 (*p* value = 0.257 FDR corrected *p* = 0.538)	r = 0.047982 (*p* value = 0.781 FDR corrected *p* = 0.890844)	r = 0.339916 (*p* value = 0.042 FDR corrected *p* = 0.155)	-
	**Steps/day**	-	-	-	-	-	-	-	-	-	-	Estimate (95% CI): 0.3 (−370, 371) *p* value = 1.00 Standardized β\betaβ (95% CI): <0.01 (−10.0, 10.0)

r: Pearson’s coefficient; FDR corrected *p*: the false discovery rate corrected *p* value; ESS: Epworth Sleepiness Scale; RBDSQ: Rapid eye movement sleep Behavior Disorder Screening Questionnaire; REM: rapid eye movement; and NREM: non-rapid eye movement. In bold: significant association between variables.

## Data Availability

Not applicable.
